# An Exploration of Nanoparticle-Based Diagnostic Approaches for Coronaviruses: SARS-CoV-2, SARS-CoV and MERS-CoV

**DOI:** 10.3390/nano12203550

**Published:** 2022-10-11

**Authors:** Ahmed Al-Hindawi, Usama AlDallal, Yousef Mostafa Waly, Muhammed Hesham Hussain, Mohamed Shelig, Omar Samir Mohamed Megahed Saleh ElMitwalli, G. Roshan Deen, Fryad Z. Henari

**Affiliations:** School of Medicine, Royal College of Surgeons in Ireland (RCSI), Medical University of Bahrain, Adliya P.O. Box 15503, Bahrain

**Keywords:** nanoparticles, coronaviruses, diagnostic techniques, COVID-19, SARS-CoV-2, MERS-CoV, SARS-CoV

## Abstract

The wildfire-like spread of COVID-19, caused by severe acute respiratory syndrome-associated coronavirus-2, has resulted in a pandemic that has put unprecedented stress on the world’s healthcare systems and caused varying severities of socio-economic damage. As there are no specific treatments to combat the virus, current approaches to overcome the crisis have mainly revolved around vaccination efforts, preventing human-to-human transmission through enforcement of lockdowns and repurposing of drugs. To efficiently facilitate the measures implemented by governments, rapid and accurate diagnosis of the disease is vital. Reverse-transcription polymerase chain reaction and computed tomography have been the standard procedures to diagnose and evaluate COVID-19. However, disadvantages, including the necessity of specialized equipment and trained personnel, the high financial cost of operation and the emergence of false negatives, have hindered their application in high-demand and resource-limited sites. Nanoparticle-based methods of diagnosis have been previously reported to provide precise results within short periods of time. Such methods have been studied in previous outbreaks of coronaviruses, including severe acute respiratory syndrome-associated coronavirus and middle east respiratory syndrome coronavirus. Given the need for rapid diagnostic techniques, this review discusses nanoparticle use in detecting the aforementioned coronaviruses and the recent severe acute respiratory syndrome-associated coronavirus-2 to highlight approaches that could potentially be used during the COVID-19 pandemic.

## 1. Introduction

### 1.1. Global Prevalence

In December 2019, a new strain of coronavirus, called severe acute respiratory syndrome-associated coronavirus-2 (SARS-CoV-2), was identified in Wuhan, China [1]. The virus has led to the rapid outbreak of an infectious disease known as coronavirus disease 2019 (COVID-19). As of 2 August 2022, the disease has infected more than 580 million people and led to at least 6.4 million deaths globally [2].

A COVID-19 pandemic was declared by the WHO on March 11, 2020, causing governments and health agencies to take drastic measures in hopes of halting the spread of the virus, including restricting travel and nationwide lockdowns [3]. The current pandemic is considered a crisis, in which the world economy is expected to undergo severe negative and long-lived consequences [4].

### 1.2. Importance of Diagnostics in the COVID-19 Pandemic

With no specific treatments against COVID-19, the primary strategies adopted by governments have revolved around preventing the spread of the disease rather than relying on therapeutics or repurposed drugs. Such strategies include the enforcement of lockdowns (periodic shutdowns of most public amenities for 21–28 days), home quarantine and self-isolation (up to 14 days upon positive COVID-19 PCR result), social distancing, restricting social gatherings, developing low-cost detection methods with high specificity and selectivity (immunoassays and ‘test-at-home’ kits) and vaccination campaigns. Awareness campaigns on the use of personal protective equipment (PPE) and practicing vigilant personal hygiene have also been implemented [5,6].

The efficacy of such measures as home quarantine and self-isolation in slowing the spread of COVID-19 depends heavily on the rapid and accurate diagnosis of the disease. With healthcare systems worldwide being under unprecedented stress, the importance of efficient diagnostic techniques and preventive measures cannot be understated.

The current standard methods of diagnosis rely on reverse-transcription polymerase chain reaction (RT-PCR) and computed tomography (CT) [7,8]. RT-PCR entails the collection of viral RNA to detect the pathogen’s presence, while CT involves the stitching of several chest x-ray images to generate a 3-D image [9]. While accurate and effective, several disadvantages accompany RT-PCR, including the high cost of operation, lengthy sample processing, false positive or negative results and laborious handling [10,11]. On the other hand, besides exposing patients to radiation, standalone CT scanning is insufficient to provide a diagnosis as there are difficulties in precisely identifying a causative agent of pneumonia [11]. As such, CT has primarily been used as a confirmatory method for potential false-negative RT-PCR results [12,13].

With the continuous emergence of evolving variants of SARS-CoV-2, the need for economical, reliable and adaptive diagnostic methods is ever-increasing. The virus’s variants have introduced challenges in efficiently diagnosing patients, as the strain’s mutations result in increased rates of false-negatives and heterogeneity of clinical presentations [14]. Through consistent follow-up of the various SARS-CoV-2 sub-lineages, characteristic differences have been reported to influence transmission rates and disease severity [15]. For instance, the Omicron variant was seen to spread more easily amongst the general population while simultaneously having higher risk of re-infection [14]. Similarly, the Delta variant is also a significantly more contagious form of the virus [16]. Indeed, the effect of viral recombination was seen to create highly pathogenic strains with improved environmental survival fitness, leading to the reoccurring waves of COVID-19 infections [15]. Hence, adequate containment of the virus’s new variants depends heavily upon advancements of accurate, rapid, yet affordable means of diagnosis, especially in developing countries [16].

To this end, a quick, sensitive, specific and cost-effective diagnostic test for SARS-CoV-2 is urgently required. An option that would fit these criteria would be nanoparticles (NPs). The literature has documented that nanoparticles have rapidly been utilized to detect microbes, including coronaviruses. Similar to the repurposing of drugs to manage COVID-19, previous NP-based diagnostic approaches for coronaviruses have the potential to be adapted for use during the current pandemic.

With that said, this review will outline the relevant information for the detection of SARS-CoV-2 (the virus’s structure and pathogenesis) and delineate the major types of coronaviruses. Principally, this review aims to showcase and discuss NP-based endeavors for detecting coronaviruses, specifically those that caused past outbreaks like severe acute respiratory syndrome-associated coronavirus (SARS-CoV) and middle east respiratory syndrome coronavirus (MERS-CoV). Furthermore, recent developments in detecting SARS-CoV-2 using NPs will be explored.

### 1.3. Nanoparticles

Nanoparticles are defined as a group of small elemental units, collectively behaving as a singular unit, giving rise to novel properties not demonstrated or seen in the bulk material [17]. The size of a nanoparticle typically ranges between 1–100 nm [18]. At the scale of a nanometer, the unique arrangement of the atomic structure ultimately results in the portrayal of new chemical, physical and biological properties.

Overall, the unique properties of nanoparticles are largely due to their large surface areas and ability to self-assemble. The immensely small particle size and the increased proportion of surface atoms contribute to a significant increase in the surface area of active sites, leading to increased reactivity [17]. In addition, the process of self-assembly, where components are organized to produce an ordered pattern, is attributed to the individual properties of nanoparticles, including shape, charge and polarizability. Such processes would define the repulsive and attractive forces between individual nanoparticles, determining their unique sensitivity to external stimuli [17]. Furthermore, nanoparticles have been seen to contribute to lower toxicity rates while retaining greater efficacy, making them a promising tool for preventing, treating and diagnosing viral infections [19,20].

**Figure 1:**
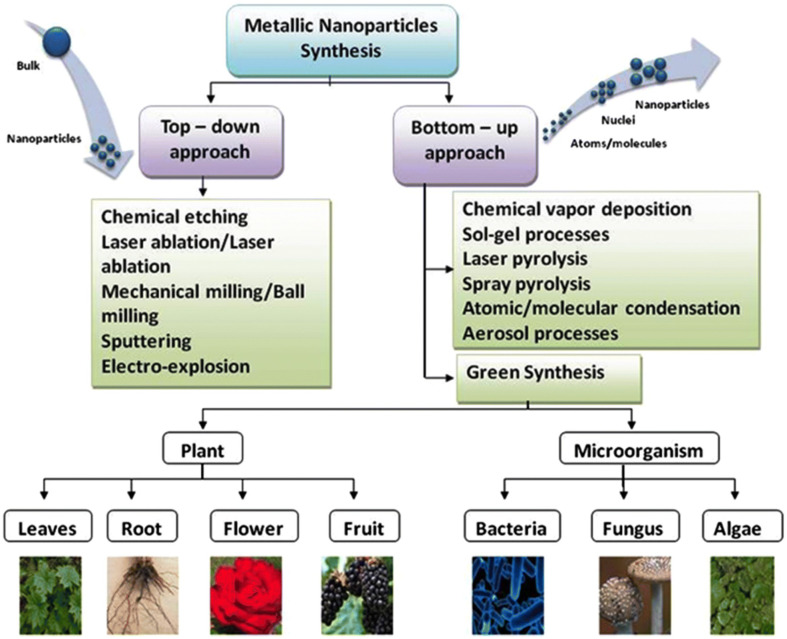
Illustration of top-down and bottom-up nanoparticle synthesis Methods. Reproduced from [28] under the Creative Commons Attribution 4.0 International License.

Many protocols have been developed to synthesize NPs using physical, chemical, or biological procedures, involving either top-down or bottom-up approaches [21] (illustrated in Figure 1). The top-down technique relies on breaking down material into nano-sized components through physicochemical means [21], such as thermolysis [22], nanolithography [23], laser ablation [24], irradiation [25,26] and sputter deposition [27]. The bottom-up technique combines and assembles atoms, molecules, or clusters to form nanoparticles using green and wet chemical synthesis methods.

However, the aforementioned physicochemical methods of NP synthesis present several disadvantages, including requirements for high amounts of temperature and pressure, production of toxic waste and the huge costs needed to initiate such processes [21]. On the other hand, biological methods use micro-organisms and plant-based compounds to produce intracellular or extracellular NPs [21]. They offer unique advantages such as being non-toxic, eco-friendly and considerably cheaper than their physicochemical counterpart [29]. Furthermore, green NPs boast rapid synthesis, high stability and increased drug conjugation potential while avoiding the need for high pressures or temperatures [30,31]. To this end, the green synthesis method has proved to be a desirable and reliable approach to generate NPs.

NPs of various compositions, such as metallic NPs, carbon-based materials NPs and quantum dots, have been widely used to diagnose viruses. More specifically, metallic NPs such as gold, silver and titanium have been seen to aid in diagnosing Ebola, HIV, influenzas and herpes simplex virus due to their encapsulation properties and optical characteristics [32,33]. Given their properties and the immense customizability of their physiological and physicochemical features, NPs are a promising tool that could be used to detect and diagnose coronaviruses [34]. Mechanisms of detection of coronaviruses using NPs vary greatly depending on the type of NP used, the desired molecular target and the apparatus that the NP is a part of. Potential mechanisms of action may include: (1) isolation of coronavirus RNA using superparamagnetic NPs; (2) aggregation-based columetric changes facilitated by gold NPs, detected through a biosensor or naked eye; (3) detection of electrochemical changes, facilitated by carbon nanostructures or gold NPs [35]. Further detail of detection mechanisms will be described in the following sections pertaining to nanoparticle application in the corresponding sections on the discussed coronaviruses.

## 2. Types of Coronaviruses

### 2.1. Severe Acute Respiratory Syndrome-Associated Coronavirus (SARS-CoV)

SARS, otherwise known as severe acute respiratory syndrome, is a disease that first emerged in 2002 in Guangdong Province, China. It had infected approximately 8100 cases and resulted in 800 deaths with a mortality rate of 9% by 2003 [36]. The disease is driven by Severe Acute Respiratory Syndrome Coronavirus (SARS-CoV) [37]. The virus is thought to have originated from bats, infecting humans through zoonotic transmission [38]. The main method of dissemination has been speculated to be through human–human close contact via aerosols [39]. SARS-CoV is a single-stranded RNA (ssRNA) virus [39] that binds to angiotensin-converting enzyme receptor 2 receptor expressed in many epithelial tissues, primarily in the lungs and gastrointestinal tract and other organs, including the heart and kidneys [39]. Due to the extensive expression of ACE2 receptor in the lower respiratory tract [39], the clinical presentations of SARS-CoV patients typically mimic that of SARS-CoV-2, which include, but are not limited to, fever, lethargy, myalgia and dry cough [36].

### 2.2. Middle East Respiratory Syndrome Coronavavirus (MERS-CoV)

MERS, also known as Middle Eastern respiratory syndrome, is a disease that originated in Saudi Arabia in 2012, infecting over 2500 patients and resulting in the fatality of approximately 870 [36]. MERS-CoV is an ssRNA virus that binds to dipeptidyl peptidase-4 (DPP4), a receptor expressed on endothelial cells, macrophages and fibroblasts [40]. The disease and symptomatology are caused by Middle Eastern respiratory syndrome associated-coronavirus (MERS-CoV), which is thought to have originated from camels through zoonotic transmission [41]. The number of infections has noticeably decreased over the years due to the readily available diagnostic methods in more recent times compared to SARS-CoV [36]. The primary symptoms of MERS-CoV infection include fever, dyspnea, dry cough and myalgia [40].

## 3. Structure and Pathogenesis of SARS-CoV-2

**Figure 2:**
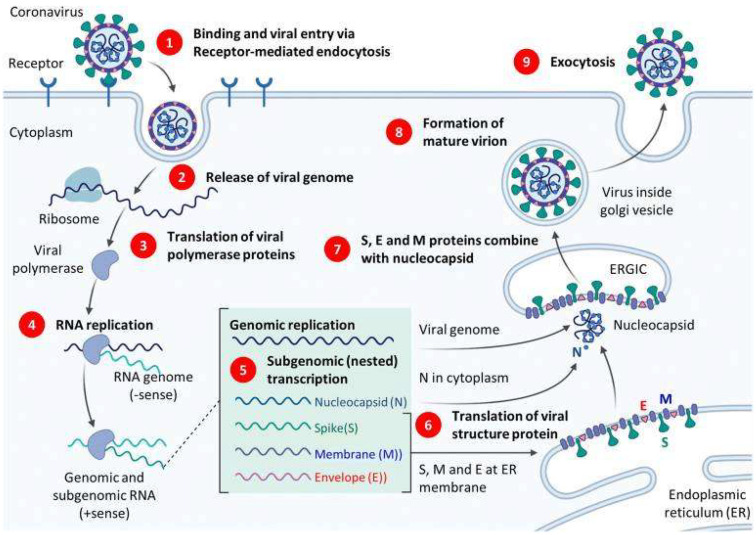
Illustration of SARS-CoV-2 replication cycle. Reproduced from [43] under the Creative Commons Attribution 4.0 International License.

Our virus of interest, SARS-CoV-2, is a single-stranded RNA coronavirus made of approximately 26–32 kilobases and is covered with a helical-like nucleocapsid that protects its genome [36]. The main components that preserve its structural integrity include the spike (S) glycoprotein as well as the membrane (M), E (envelope) and the N (nucleocapsid) proteins [36]. The N and E proteins are expressed by the N and E RNA [42]; they are the nucleic acid targets of detection in RT-PCR [9]. An illustration of the replication cycle of SARS-CoV-2 and relevant RT-PCR targets can be seen in Figure 2.

It is noteworthy to mention that SARS-CoV-2′s genome is approximately 82% identical to the original 2002 SARS-CoV and the 2012 MERS-CoV, with >90% similarity in sequences coding for essential enzymes and structural proteins [44]. Therefore, it is essential to examine the previous diagnostic approaches for both viruses and attempt to translate them to a SARS-CoV-2 diagnostic approach.

The Spike S protein is the most extensively studied antigen. This protein binds to the angiotensin-converting enzyme 2 (ACE2) receptor, a receptor generally expressed in epithelial linings, such as respiratory and gastrointestinal tracts and organs such as the heart, liver and kidneys [39]. The S protein has two subunits, S1 and S2. Within the S1 subunit is a domain named the receptor-binding domain (RBD), responsible for binding to the ACE-2 binding domain [45]. The S2 Subunit is essential for membrane fusion [45]. The viral RNA then translocates and integrates within the host cell’s DNA, utilizing its replication machinery to transcribe the viral mRNA. The mRNA is then translated to proteins that become2 assembled to new replicated SARS-CoV-2 and subsequently released [45]. One of the important genes involved in replication and transcribing of the viral mRNA is the RNA-dependent RNA polymerase (RdRp) gene. This gene, along with the N and E genes, is also used to detect SARS-CoV-2 [9].

ACE-2 receptors are highly expressed in the epithelium of lungs, including bronchial and bronchial branches’ epithelia [46]. As a result, the symptoms of SARS-CoV-2 invasion are clinically presented as pneumonia and acute respiratory distress syndrome [47]. ACE-2 receptors are also expressed in the kidneys, heart and liver [48]. Therefore, SARS-CoV-2 infection can systematically spread and, in severe cases, result in multiple organ failures [47]. In the cardiovascular system, the binding of the virus to the ACE-2 receptor activates signal transduction pathways, including the as-ERK-AP-1 pathway that activates pro-fibrosis factor, resulting in the development of cardiac inflammation and fibrosis [49].

Mutations to the aforementioned molecular targets have3333 led to increased pathogenicity and detection alterations of the various SARS-CoV-2 variants. Regarding the Omicron variant, a deficiency in spike cleavage, resulting in inefficient transmembrane protease-serine 2 (TMPRSS2) usage, allows for a dramatic increase in replication within the lungs [50]. As a result, RT-PCR can be set to search for a S gene target dropout, facilitating faster detection rates [51]. Conversely, the Delta variant’s increased contagiousness is attributed to an increased propensity for more RBD-up states and an affinity-enhancing T478K substitution, both of which increase ACE2 receptor binding [52]. The subsequent enhances replication allows for earlier detection, where the Delta variant has an average detection time of 3.2 days from exposure compared to the 4.5 days of the early alpha variant virus [53].

## 4. Current Methods of COVID-19 Diagnosis

Numerous methodologies have been applied to detect COVID-19. Currently, COVID-19 is primarily diagnosed using three techniques: (1) reverse-transcription polymerase chain reaction (RT-PCR) and gene sequencing, (2) chest computed tomography (CT) and (3) lateral flow immunoassay [7,8].

### 4.1. Reverse-Transcription Polymerase Chain Reaction (RT-PCR)

The current gold standard for diagnosing COVID-19 is RT-PCR due to its high selectivity and sensitivity [9,54,55,56,57], providing ~95% accuracy under ideal conditions [58]. In order to perform RT-PCR, a biological fluid sample containing SARS-CoV-2 strains is collected. A sample would usually entail upper and lower respiratory fluid collected using nasopharyngeal and oropharyngeal swabs [9]. Subsequently, the collected fluid would undergo filtration and separation steps to isolate the viral RNA, from which complementary viral DNA (cDNA) is created [59]. A large number of cDNA (proportional to the concentration of the virus) would generate a sizeable fluorescent signal following several rounds of polymerase reaction [59]. If the system is well-calibrated, fluorescence intensity would directly reflect the concentration of the virus within an infected patient [59]. Until now, three regions of the cDNA have been identified for detection of SARS-CoV-2: E, N and RdRP genes [9].

Despite its high selectivity and sensitivity, RT-PCR is accompanied by numerous challenges and disadvantages. The technique requires complex and expensive equipment and a laboratory with biosafety level 2 or above [60]. In addition, further prerequisites, including technically skilled personnel and a stable power supply (9), hinder the use of RT-PCR in several COVID-19 outbreak regions where there is a lack of infrastructure. The method takes 3–6 h from sample collection to result generation [10]. However, due to the sheer number of requested tests, the time required to obtain the results can be up to 2–3 days [60]. The massive amounts of reagents needed to perform the testing have also become a bottleneck [61]. Such inefficient systems can be detrimental to public health safety, especially since we live during a COVID-19 crisis. Furthermore, the wide variability of viruses in different patient samples has given rise to false-negative results [62]. The relatively low sensitivity of RT-PCR if patient samples are not of high purity (blood samples or sputum) can also explain the emergence of false-negative results [63,64]. Lastly, isolated RNA degrades rapidly and requires immediate freezing [59]; poor handling of samples may be another reason for false-negative results. All the factors mentioned above contributing to lower sensitivities can be exemplified by the sensitivity of the first RT-PCR test for SARS-CoV-2 being only 70% [65]. Consequently, CT is being used in combination with RT-PCR as a confirmatory diagnostic measure for patients with clinical suspicion who gave a negative RT-PCR result [12,13].

### 4.2. Computer Tomography (CT) Scan

CT scan is currently being used as either a standalone diagnostic tool or alongside RT-PCR (to ascertain negative results in symptomatic patients) in diagnosing COVID-19 [12,13]. In the context of COVID-19, CT revolves around taking many chest X-ray measurements from different angles. These measurements would then be compiled to create a three-dimensional (3D) image with contrast; such images are investigated by radiologists. Characteristic COVID-19 presentations that appear in CT include “areas of subpleural regions of ground glass opacification affecting the lower parts of either a single lobe or both lobes” [59]. CT scans can further be used to assess the prognosis of a patient with COVID-19. For instance, within the first few days of the infection, the image mimics a regular chest CT. However, as the disease progresses, the ground-glass-like opacity becomes more pronounced with bilateral peripheral predominant consolidation [66].

One of the main challenges that radiologists face is attempting to differentiate the symptoms of COVID-19 from other lung conditions, especially other pneumonia-causing pathologies [59]. Indeed, CT scans are reported to have a specificity of detection of approximately 25%, mainly due to the imaging characteristics and signs sharing significant similarities with other viral pneumonia [67]. Nonetheless, as CT scans are expensive and require knowledgeable personnel with high technical skills to operate and interpret, it has been chiefly used as a complementary method in SARS-CoV-2 detection [12,13].

### 4.3. Rapid Antigen Testing (RAT)

As an alternative to RT-PCR and radiological imaging, healthcare centers, particularly those in developing nations or rural regions, have been employing RAT kits to quickly determine the clinical management of symptomatic patients [68]. Using immunochromatography, the method revolves around the interaction between desired antigens (typically SARS-CoV-2 nucleocapsid protein) and antibodies implanted onto nitrocellulose membranes [69]. Results can be interpreted either through immunofluorescence or with the naked eye, through the presence of a colored band. RAT offers unique advantages including being an inexpensive, rapid and intuitive test that could be performed in a point-of-care setting or by patients themselves. Yet its main drawback is relatively poorer sensitivity and selectivity when compared to RT-PCR [68]. Indeed, the WHO recommends that a RAT kit should have a sensitivity of 80% and a specificity of 97% (compared to RT-PCR) to be clinically appropriate [69]. While such performance markers vary between RAT kit types and manufacturers as well as the included patient selection, a meta-analysis conducted by Khandker et al. involving 17,171 suspected COVID-19 patients reported a lack-luster sensitivity of 68.4% (95% CI: 60.8–75.9) and specificity of 99.4% (95% CI: 99.1–99.8). Further, the reported sensitivity of RAT kits within asymptomatic patients was 54.5% [69]. To this end, negative RAT results are not enough to rule out a COVID-19 infection and further molecular investigation may be indicated, especially for symptomatic patients.

### 4.4. Immunoassays and Enzyme-Linked Immunosorbent Assays (ELISA)

COVID-19 has been diagnosed through the detection of anti-SARS-CoV-2 IgG in serum. A prominent type of this technique is enzyme-linked immunosorbent assay (ELISA). Through the use of microtiter plates, such as the 96-well, antibodies are detected through protein–protein interactions [59]. A signal is then detected via fluorescence, luminescence, or colorimetric techniques [59]. The applications of this procedure can go beyond diagnosis. Changes in serum SARS-CoV-2 IgG levels can be monitored, providing a way to evaluate treatment response and prognosis [70]. Furthermore, the presence of antibodies can confidently confirm whether vaccines are effective [59]. It can also be an invaluable procedure in aiding intervention policymakers on the number of asymptomatic patients within a population [59].

Within diagnosis, immunoassays have the potential to provide a result very rapidly. A recent report has described the performance of a rapid test based on IgM and IgG; compared to RT-PCR, it showcased a lower sensitivity of 86.66% [60]. Nonetheless, the main advantage of using immunoassays remains in their remarkable speed in providing a result.

Challenges associated with immunoassays revolve around their sensitivity, technical issues and the time required to produce antibodies since the onset of infection. To begin, there are potential difficulties with creating an accurate serological test that can precisely differentiate between SARS-CoV-2 antibodies and antibodies generated against other coronaviruses [59]. As such, false-positive results may emerge due to a lack of selectivity between different coronaviruses’ antibodies. Interestingly, current antibody tests have been reported to present false-negative results. The majority of false-negative results are said to be caused by the following technical issues: “(1) a low concentration of antibodies typically present in fluidic samples; (2) presence of homologous proteins; and (3) lack of sensitivity from the detection instrument” [59]. Perhaps the most critical disadvantage of immunoassay lies in the fact that IgM and IgG are typically detectable two weeks after the onset of the infection (8). As a result, early detection of SARS-CoV-2 using immunoassays is difficult, forcing health workers to rely on other diagnostic methods such as RT-PCR and CT. Other issues pertaining to immunoassays, specifically ELISA, include labor-intensive sample collection and long incubation times [71].

### 4.5. Loop-Mediated Isothermal Amplification (LAMP)

Developed by Notomi et al. in 2000, LAMP has garnered interest in diagnostics due to its quick, sensitive and ultimately effective amplification method using nucleic acids [72]. Detection can be seen visually using intercalating fluorescent dyes [72] or colorimetric techniques [73]. As it is notably cheaper to operate than RT-PCR and still able to detect nucleic acids, isothermal thermal amplification methods are being developed to overcome RT-PCR’s cost and resource [74]; the method presents advantages that can be utilized in POC testing [74]. In some instances, LAMP has been reported to be faster and marginally more sensitive than conventional PCR methods [62]. Consequently, the technique has been deemed appropriate for detecting viruses such as MERS-CoV, SARS-CoV and influenza A [62].

LAMP has been implemented alongside RT (RT-LAMP) to provide a simple and high-throughput method of detecting SARS-CoV-2. The detection time was reported to be 30 min, highlighting its potential in POC and screening tests [74]. Furthermore, LAMP’s efficiency in mass detection can be further amplified as it is possible to use unpurified samples in conjunction with colorimetric detection [74].

However, LAMP still has its share of disadvantages. It has been reported that LAMP is less sensitive than PCR in cases of complicated samples, such as blood. The presumed reason for this is its use of Bst DNA polymerase, while PCR employs Taq polymerase [75]. In addition, the suitable development of primers is a limitation associated with LAMP [75]. Additional research on the stability and consistency of LAMP is required for its widespread application in clinical diagnosis [76].

## 5. Usage of Nanoparticles in the Detection of Coronaviruses

Nanoparticle-based biosensor prototypes illustrate desirable diagnostics qualities, especially during the current pandemic. Such qualities include their ease of use, inexpensive equipment and visual results [77]. Gold nanoparticles (AuNPs) have fostered widespread attention in the past decade due to their outstanding optical characteristics, photostability and high extinction coefficient [77]. The extinction coefficient is controlled by particle size, shape and the local refractive index near the particle surface.

In the following sections, we will discuss the previous applications of various types of nanoparticles in detecting coronaviruses. Different detection techniques have been utilized, including electrochemiluminescence, photoluminescence, colorimetric sensing and immuno-sensing [78]. Generally, the usage of nanomaterials within this field of study has been reported to decrease the time required for detection while also increasing sensitivity [78], showcasing the potential for designing improved detection approaches. As there is a pressing demand for rapid, sensitive, easily operated and affordable methods for detecting SARS-CoV-2, the benefits of nanoparticles can especially be applied during the COVID-19 pandemic.

### 5.1. Usage of Nanoparticles in the Detection of Middle East Respiratory Syndrome Coronavirus (MERS-CoV)

MERS-CoV was first reported in Saudi Arabia in September 2012, with further investigations suggesting that the first cases occurred in Jordan in April 2012 [36]. Like COVID-19, patients developed severe respiratory illness accompanied by fever, cough and shortness of breath. The mortality rate was approximated 30–40% [36].

Over the last few years, major strides have been made in MERS-CoV’s rapid and accurate detection using nanoparticles, especially AuNPs. AuNPs have been implemented both as the sole mechanism of detection or merged with established methods to improve detection rates. Through a competitive electromechanical immunosensor, Layqah and Eissa [79] used recombinant spike protein S1 (from spiked nasal samples) as a biomarker for MERS-CoV. The competition that occurred on the device was between the free viruses in the sample and the immobilized MERS-CoV S1 protein in the presence of known concentrations of antibodies [79]. The immunosensor was based on an array of carbon electrodes modified with an AuNP coating, with the voltametric response being the signal for detection. AuNPs were employed to increase the surface area, the transfer rate of the electrode’s electrons and ultimately, the peak current of the cathode [79]. The immunosensor was able to complete the analysis in 20 min while maintaining a LOD of 1.0 pg/mL and a high level of selectivity for MERS-CoV. Compared to the conventional methods used during that period, the immunosensor’s LOD was lower than the reported ELISA’s LOD of 1 ng/mL; the authors attributed the superior sensitivity to the AuNPs-modified electrodes [79].

**Figure 3:**
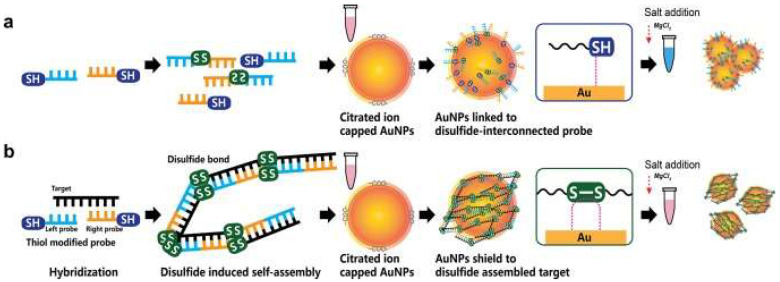
Disulfide bond-based colorimetric assay developed by Kim et al. for MERS-CoV detection. The color change is determined by whether the gene target is absent (**a**) or present (**b**). Repurposed from [80].

Furthermore, AuNPs have been incorporated in colorimetric hybridization assays. The disulfide bond-based colorimetric assay developed by Kim et al. [80] for MERS-CoV is an example of one such assay. Using an extended form of “double-stranded DNA (dsDNA) self-assembly shielded” AuNPs, they could detect the presence of MERS-CoV viral molecules. The mechanism of detection involved color changes and the shift of a localized surface plasmon resonance (LSPR) in the visible range [80] (shown in Figure 3). The thiolated single-stranded DNA probes would identify specific regions of the MERS-CoV genome, genes upstream of the E protein gene and Open Reading Frame 1a to form a self-assembled complex. The complex would protect the nanoparticles from salt-induced clumping. Thereby, in the absence of the MERS-CoV targets, the AuNPs would aggregate together, resulting in a color change. The assay was reported to provide a result within 10 min; the naked eye would easily see the result. Further, with a potential detection limit of 1 pmol/µL, it can detect the coronavirus with little-to-no amplification [80]. Such assay would be of great use in high-demand and resource-limited sites.

To accelerate detection and avoid the requirement of specialized facilities, flow detection strips utilizing NPs have been developed. By using a combination of vertical flow (VF) detection and reverse transcription loop-mediated isothermal amplification (RT-LAMP-VF), the N gene of MERS-CoV was visually detected [81]. MERS-CoV RNA was amplified and the resultant amplicons were marked with biotin and fluorescein isothiocyanate (FITC), allowing them to bind streptavidin-AuNPs conjugates and form complexes. This complex would then be targeted by anti-FITC antibodies, producing a visible colored line [81]. Considering preparation time and the time required to present a result, the test was reported only to take 35 min. The RT-LAMP-VF assay was able to detect 1 × 101 copies/µL of MERS-CoV RNA, which was more sensitive than the traditional RT-LAMP (1 × 102 copies/µL) but less sensitive than RT-PCR (1 × 100 copies/µL) [81]. The assay also displayed a high degree of specificity when evaluated with other coronaviruses, including SARS-related (SARSr)-CoV, HKU4, HKU1, OC43 and 229E [81]. The assay’s rapidity, good sensitivity/selectivity and, most importantly, lack of any need for expensive equipment make it a promising option that can be adapted in various laboratory settings.

Like AuNPs, silver nanoparticles (AgNPs) have been explored in the detection of MERS-CoV. Teengam et al. [82] developed a multiplex paper-based colorimetric sensor to detect infection-associated DNA using AgNPs as the colorimetric agent. The analytical device was tested to screen MERS-CoV and other microbes such as Mycobacterium tuberculosis and human papillomavirus. Using pyrrolidinyl peptide nucleic acid probes as opposed to DNA or RNA probes, the team measured the color change associated with AgNPs dispersion, giving a detection limit of 1.53 nM for MERS-CoV [82]. Moreover, the probe displayed high selectivity for the desired oligonucleotides, even when introduced to single-base mismatch, two-base mismatch and non-complementary DNA targets [82]. The multiplex was also described as capable of providing accurate detection for simultaneous testing of multiple DNA targets within a single device, simplifying analysis when compared to conventional techniques [82].

The use of NPs in detecting MERS-CoV is not limited to metal NPs such as AuNPs and AgNPs. Indeed, NPs like bacteria-based nanobioparticles have shown promise in the field of diagnosis. Through the biosynthesis of NPs (MERS nucleoprotein) inside Staphylococcus Aureus cells, Qioa et al. [83] were able to detect viral antibodies against MERS-CoV. MERS nucleoproteins were fabricated using a cell wall binding domain (CBD) from a bacteriophage lysin PlyV12 [83]. An agglutination test was performed to detect IgG using the Staphylococcus Aureus nanobioparticles. The agglutination was credited to the reaction between the IgG antibodies of MERS nucleoprotein, protein A on the surface of a staphylococcal particle and the MERS nucleoprotein-CBD adhered to another particle [83]. The specificity of this approach was tested by introducing the sensitized nanobioparticle to 38 clinical sera. No agglutination was observed in all 38 clinical sera and the nanobioparticle was deemed to have appropriate specificity. As the approach can provide a result within 20 min, this novel incorporation of NPs can provide an avenue for developing simple agglutination tests without unique instrumentation or expertise [83].

### 5.2. Usage of Nanoparticles in the Detection of Severe Acute Respiratory Syndrome-Associated Coronavirus (SARS-CoV)

Identified in 2003 in China, SARS-CoV was responsible for the 2002–2003 epidemic that infected 8000 people in 26 countries. It led to the death of 774 people [36]. A patient with SARS typically presents with fever, dry cough and diarrhea within the first or second week of infection. Often, the disease would progress and a patient’s condition would worsen rapidly, resulting in respiratory distress and intensive care management [36].

With the wide range of applicability and the unique optical qualities AuNPs offer, their use in SARS-CoV detection has been previously studied. Park et al. [84] effectively utilized AuNPs’ optical properties to detect SARS-CoV. Using a gold-binding polypeptide (GBP) as a fusion agent, the SARS-CoV E protein was immobilized on AuNPs. These AuNPs would then be modified with a green fluorescent protein and adhered to a bare gold surface to generate a nanopattern. The AuNP-E protein complex would demonstrate changes in both absorbance and color when exposed to its complementary antibody. These changes were recorded and subsequently used to determine the presence of SARS-CoV. The authors elaborate on how such a mechanism can create effective and high-throughput bioassays when combined with surface plasma resonance and microfluidic channels [84]. AuNPs’ characteristics were also exploited by Huang et al. [85], where they were utilized in a localized surface plasmon coupled fluorescence (LSPCF) fiber-optic biosensor. The biosensor’s mechanism of detection involved monitoring the fluorescence of a fluorophore-labeled anti-SARS-CoV N protein [85]. The device exhibited a detection sensitivity of 1 pg/mL for N protein in human serum. A linear response was demonstrated between the fluorescence signal and concentration of N protein from 0.1 pg/mL to 1 ng/mL. When compared with the conventional ELISA, the detection limit of the LSPCF fiber-optic biosensor was shown to be increased by 104-fold. The device’s very high sensitivity, quantitative ability, ease of use and disposability make it a desirable option for the early diagnosis of SARS infection [85].

**Figure 4:**
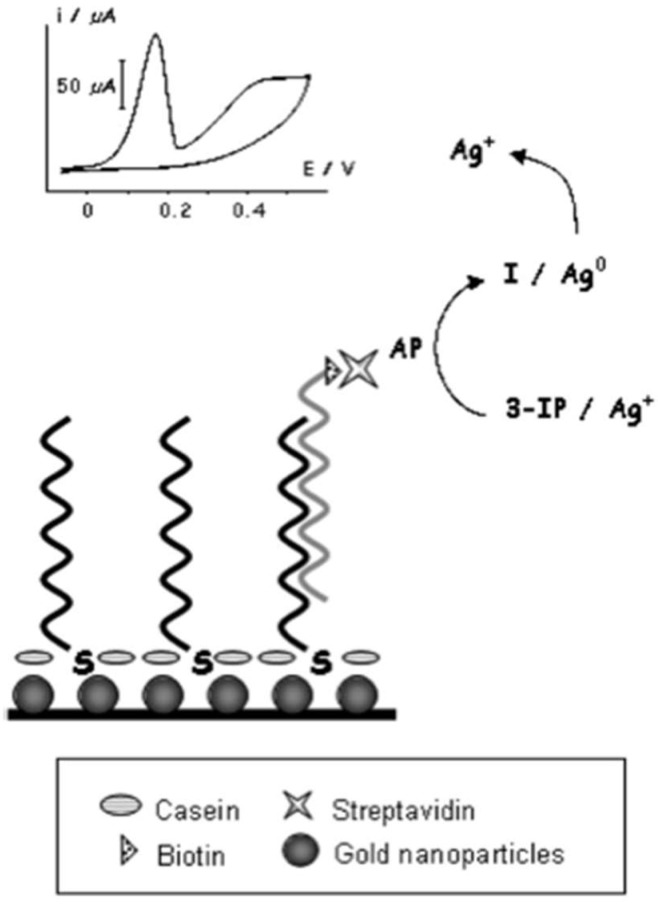
Basic schematic representation of the genosensor device developed by Martinez-Paredes et al. [86].

NP-based hybridization assays for the detection of SARS-CoV have also been researched. An electrochemical hybridization assay based on AuNP has been described using a gene-based biosensor [86]. The gene-sensor (illustrated in Figure 4) consists of immobilized oligonucleotide probes on disposable AuNP-modified screen-printed carbon electrodes; the probes hybridize the biotinylated target DNA of SARS-CoV [86]. The catalyzation of the reduction of silver (Ag) ions and subsequent deposition of metallic Ag on the electrode’s surface would follow. The amount of metallic Ag can be quantified, which is directly proportional to viral DNA load, and a result can be reported within 20 min. The sensor’s selectivity was assessed by measuring analytical signals when hybridization was conducted using complementary strands 1, 2 and 3-base mismatch. Under 25% formamide, the sensor was able to discriminate between the complementary strands and the mismatched strands. Furthermore, a linear sensor response was reported between 2.5 to 50 pmol/L, suggesting that 2.5 pmol/L was the sensor’s detection limit. The sensor’s sensitivity was also described to be greater than that achieved by surfaces without NPs [86].

Carbon nanotube (CNT) field-effect transistors and biosensors hold great potential in diagnostics owing to their mechanical durability and high thermal conductivity. In addition, single-walled CNTs have the smallest diameter compared to other one-dimensional nanomaterials, making them a remarkable option in terms of sensors, as every atom in the CNTs is in contact with its environment [87]. To study the relationship between CNT density and sensitivity, Ishikawa et al. [87] tested their density-optimized CNT biosensors to detect the N protein of SARS-CoV under physiological conditions. The device successfully detected the biomarker protein with a detection limit of 5 nM [87].

NPs, such as superparamagnetic NPs, can be used to capture targeted viral genetic material from a mixture of numerous genetic molecules for amplification. To this end, Gong et al. [88] designed functionalized silica-coated superparamagnetic NPs to isolate the desired cDNA of SARS-CoV from a sample that contains the target cDNA and non-target cDNA. The isolated cDNA was then amplified through a general symmetry PCR. Next, the cDNA was once again separated from the PCR products using another batch of the same NPs [88]. Lastly, through fluorescence, the functionalized NPs were to be used as signaling probes with a sandwich hybridization method. The approach was able to detect the cDNA with a detection limit of 2.0 × 10^3^ within 6 h [88].

### 5.3. Usage of Nanoparticles in the Detection of Severe Acute Respiratory Syndrome-Associated Coronavirus-2 (SARS-CoV-2)

As the cause behind the COVID-19 pandemic of 2020, the rapid and accurate detection of SARS-CoV-2 has been a priority for researchers globally. While RT-PCR and CT have been the most used diagnostic methods thus far, NP-based diagnostic techniques are being actively developed and optimized to provide utility in the clinical setting.

Similar to how AuNPs found application in detecting MERS-CoV and SARS-CoV, it was only inevitable that they would also be used to detect SARS-CoV-2. Many of the current diagnostic tests are unreliable when tested against a viral load at its early stages of infection or a viral gene that has mutated during its spread. To this end, Moitra et al. [77] have attempted to overcome such challenges by designing an AuNP-based colorimetric assay that can directly target the SARS-CoV-2 N gene at multiple gene positions simultaneously. The AuNPs used in this biosensor are labeled with thiol-modified antisense oligonucleotides (ASOs) that specifically target the N gene of SARS-CoV-2 [77]. In the presence of the N gene, these AuNPs would aggregate, displaying a change in their surface plasmon resonance (SPR). This agglomeration is further enhanced by the addition of RNaseH, resulting in the precipitation of the AuNPs, which are visible to the naked eye [77]. The test’s limit of detection was reported to be 0.18 ng/µL. Additionally, the selectivity was also assessed by introducing the Au-ASO nanoparticles to RNA isolated from cell lysate of MERS-CoV infected Vero cells, where there was no significant change in the absorbance, making the test rather selective.

Similarly, Kumar et al. [89] developed a colorimetric nanoparticle-based assay to detect the presence of the RdRp gene of SARS-CoV-2. Through the usage of AuNPs, a pink-to-blue color change can be appreciated upon the formation of the oligo probe-target complex within assays containing SARS-CoV-2 pharyngeal RNA samples. The assay would maintain its pink color when introduced to samples from COVID-19-negative patients or human papillomavirus-infected women. When tested against RT-PCR, the developed instrument had a sensitivity of 85.29% and a specificity of 94.12%. Lastly, the detection limit was 0.5 ng of SARS-CoV-2 RNA and results can be obtained within 30 min [89]. As such, these studies exemplify how NPs can be utilized to develop a visual, reliable and reproducible COVID-19 diagnostic test without the need for sophisticated equipment.

AuNPs have also shown promise in providing extremely rapid detection (≤2 min) of SARS-CoV-2 RNA, bypassing the need for PCR amplification. Li et al. [90] designed a graphene field-effect transistor sensor studded with AuNPs, on which complementary phospho-rodiamidate morpholino oligos probes were seeded to target the RdRp gene. Through the usage of a semiconductor characterization system and a probe station, electrical changes on the sensor chip can be monitored. When tested on 30 clinical samples (20 confirmed COVID-19 patients and 10 healthy controls), a statistically significant difference in the average levels of the sensor’s signal was reported. The limit of detection was determined to be 2.29 fM for throat swabs and 3.99 fM for serum. Furthermore, a Kappa test was performed to scrutinize the reliability of the developed method and the gold-standard RT-PCR, with a result of 0.92, suggesting near-perfect agreeability. Such a tool could be adopted in the emergency setting or close-contact investigations as a screening tool, given its ability to provide rapid and reliable results [90].

By combining the plasmonic photothermal (PPT) effect and localized surface plasmon resonance (LSPR) sensing transduction, Qiu et al. [91] were able to create a dual-functional plasmonic biosensor based around gold nano-islands (AuNIs) to detect SARS-CoV-2 genetic material. These AuNIs were functionalized by forming bonds with the complementary receptors of the ORF1ab, RdPd, or E gene sequences, allowing for their sensitive detection through nucleic acid hybridization [91]. To maximize the device’s sensitivity, heat was generated by exposing the AuNIs to their plasmonic resonance frequency. Sensing stability and reliability were further improved by utilizing two different angles of incidence, permitting the use of two varying wavelengths to excite the plasmonic resonances of PPT and LSPR [91]. Consequently, the biosensor’s detection limit was reported to be 0.22 pM while simultaneously differentiating the desired targets from a multigene mixture [91]. This technique could enable clinicians to generate a reliable result in real-time without labeling the desired gene sequences.

**Figure 5:**
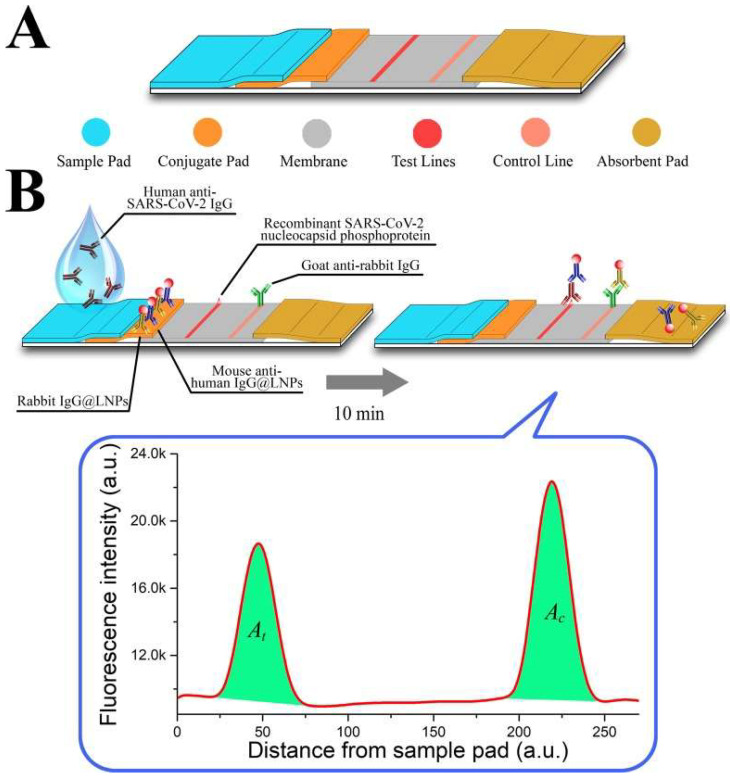
(**A**) An illustration of the developed lateral flow strip; (**B**) An illustration of the assay, incorporating the LNPs as fluorescent signalers [92].

Along with the detection of viral DNA/RNA, NPs have been developed to detect anti-SARS-CoV-2 IgG in human serum. In the lateral flow immunoassay (LFIA) created by Chen et al. [92], lanthanide-doped polystyrene nanoparticles (LNPs) were used to detect anti-SARS-CoV-2 IgG; mouse anti-human IgG marked with LNPs functioned as a fluorescent signaler (portrayed in Figure 5). The assay was tested with 100-µL aliquots of human serum samples that were diluted 1000-fold; the test’s duration was reported to only take 10 min [92]. Moreover, the diagnostic accuracy of the assay was evaluated by testing it against samples that were previously tested with RT-PCT, seven of which were positive and 12 were negative but clinically suspicious [92]. The assay was able to accurately replicate the results of the RT-PCT, giving a positive result for all seven samples. In fact, the assay also reported a positive result in one of the clinically suspicious samples. Unlike the other suspected samples, this specific case presented with relevant symptoms in addition to fever, justifying the high clinical suspicion [92]. Chen et al. showcased how an assay based on NPs can be used to ascertain the results provided by RT-PCT, provide a rapid diagnosis and also be applicable in monitoring a patient’s response to treatment.

Immunoassays revolving around AuNPs were also developed for the detection of SARS-CoV-2. Wen et al. [71] described the creation of a point-of-care LFIA used to detect the IgG antibodies against SARS-CoV-2. The strip was compromised of an immobilized SARS-CoV-2 nucleocapsid protein on the strip’s surface and anti-human IgG coupled with colloidal AuNPs. Colloidal AuNPs were mainly chosen for this sensor due to their stability, ability to provide interpretable results with the naked eye and excellent biocompatibility [71]. The detection mechanism is based on antigen-antibody interactions, where the presence of IgG antibodies of SARS-CoV-2 would form a complex with the anti-human-antibody-AuNP compound and subsequently migrate to the bound nucleocapsid protein (test line). Thereby, a positive result would be indicated based on whether there is a red color on the test line. In order to gauge the practicability of the assay in a clinical setting, Wen et al. applied the assay to detect SARS-CoV-2 in 55 different patient samples. The reported sensitivity was 69.1%, where 17 out of the 55 samples were not identified as positive. Nonetheless, when tested against samples from patients with severe fever with thrombocytopenia syndrome (SFTS) and avian influenza A (H7N9), there was no cross-reactivity signifying that the assay was of high specificity [71]. Each sample was repeated in triplets at intervals ranging from 1 to 3 weeks; the results remained unchanged, suggesting that the LFIA strip had exceptional stability. Due to its speed and cost-effectiveness, the authors propose that the LFIA strip can provide a preliminary result for physicians to initiate treatment in clinically suspicious patients. However, given its limitations in terms of sensitivity, physicians may need to use alternative testing methods to make a confident diagnosis. Similarly, Huang et al. [93] synthesized a colloidal gold nanoparticle-based LFIA that allows for the on-site detection of IgM antibodies against SARS-CoV-2. Detection is interpreted through an indirect immunochromatography technique. The strips were comprised of a SARS-CoV-2 nucleoprotein-coated membrane and an anti-human IgM-AuNP complex functioning as the reporting agent. The result can easily be seen as a red-colored line appearing on the test line and control lines. By testing it against serum samples of COVID-19 patients and normal individuals, the assay achieved a 100% accuracy [93]. It also demonstrated a degree of selectivity, showing no cross-reactions or alterations when tested against severe fever with thrombocytopenia syndrome virus and dengue virus [93]. Considering that the test requires 15 min and 10–20µL of serum to perform, it presents itself as a very attractive and feasible method to detect SARS-CoV-2.

**Figure 6:**
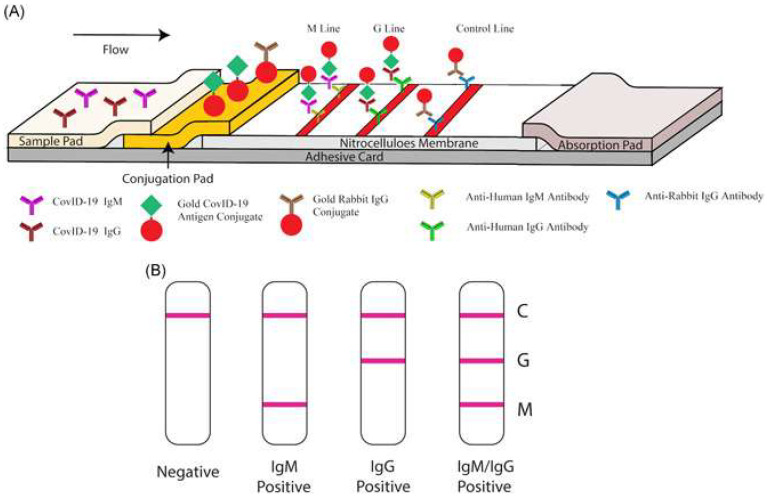
Illustration of rapid SARS-CoV-2 IgM-IgM combined antibody test. (**A**) Schematic of detection device; (**B**) Illustrations of different possible results (C: Control line; G: IgG line; M: IgM line). Reproduced from [94] under the Creative Commons Attribution 4.0 International License.

Interestingly, in an attempt to identify patients at varying infection stages, Li et al. [94] developed a point-of-care lateral flow immunoassay to detect both IgM and IgG antibodies against SARS-CoV-2 within 15 min, where the antigen is conjugated on AuNPs. Similar to the previous devices, results are communicated through detection lines on the strip. However, instead of two, three lines are used: a control (C) line, an anti-SARS-CoV-2 IgM line and an anti-SARS-CoV-2-IgG line (showcased in Figure 6). The device’s sensitivity and specificity were 88.66% and 90.63%, respectively, calculated using blood samples from 397 PCR confirmed COVID-19 patients and 128 COVID-19 negative patients. Moreover, the results were deemed reliable across sample types, whether they may be fingerstick blood or plasma of venous blood [94].

**Figure 7:**
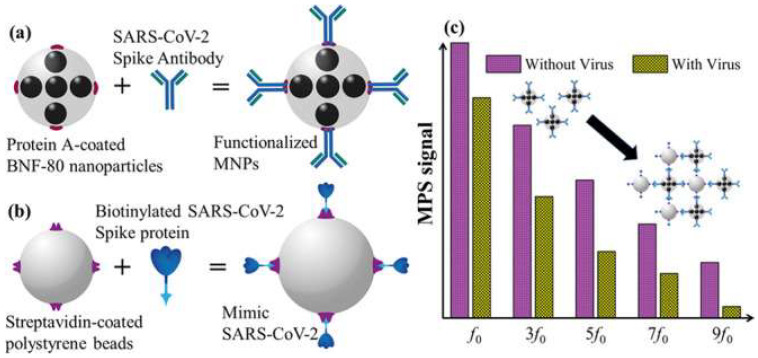
(**a**) Illustration of functionalized magnetic NPs; (**b**) illustration of SARS-CoV-2 mimic; (**c**) magnetic particle spectroscopy signal with and without SARS-CoV-2 mimic. Reprinted (adapted) with permission from Zhong et al., *Toward Rapid and Sensitive Detection of SARS-CoV-2 with Functionalized Magnetic Nanoparticles. ACS Sens.* **2021**, *6*, 976–984, doi:10.1021/acssensors.0c02160. Copyright 2021 American Chemical Society.

Other forms of nanotechnology, including graphene sheets, magnetic nanoparticles (MNPs) and nanotubular formations of conducting polymers (CPs), have been used to develop novel approaches for the detection of SARS-CoV-2. Seo et al. have developed an antibody-based biosensor functioning as a field-effect transistor (FET) to detect SARS-CoV-2 in nasopharyngeal samples [95]. The graphene sheets were coated with an antibody targeting the SARS-CoV-2 spike protein. With this approach, the device detected the protein in phosphate-buffered saline and clinical transport medium at concentrations of 1 fg/mL and 100 fg/mL, respectively [95]. When used in unprocessed clinical samples, the sensor detected the virus with a detection limit of 242 copies/mL, which could potentially be lowered by reducing noise signals. Compared to the detection limits of currently used molecular diagnostic techniques (50–100 copies/mL), the graphene-based biosensor can provide a comparable diagnostic outcome within significantly shorter periods of time [95]. Another form of applicable nanotechnology are MNPs, one of the most promising methods for sensitive biomolecule detection [96]. As an example, Zhong et al. formulated an approach to detect SARS-CoV-2 through the utilization of functionalized MNPs and the measuring of their magnetic response in an AC magnetic field [96]. As a proof of concept, the team fabricated SARS-CoV-2 replicas; each replicate is composed of 100 biotinylated spike proteins and a streptavidin-coated polystyrene bead. Using magnetic particle spectroscopy (MPS), it was seen that the binding of mimic SARS-CoV-2 particles and functionalized MNPs changed the MPS signal as it increased the effective Brownian relaxation time (illustrated in Figure 7). With a calculated detection limit of 0.084 nM (5.9 fM), the authors believe that the approach could be a promising candidate for sensitive, easy-to-perform and cheap point-of-care for rapid SARS-CoV-2 detection [96]. Lastly, nanoarchitectures of CPs, such as poly-pyrrole have been investigated for COVID-19 diagnostic applications due to their stability, ease of synthesis and electroactivity in phosphate buffer (pH 7.4) [97]. By incorporating AuNPs within poly-pyrrole nanotubular morphologies, Hryniewicz et al. added further stability, biocompatibility and selectivity to the CPs formulations, making it a promising biosensing tool for COVID-19 antibodies [97]. The authors tested two morphologies (globular and nanotubular) of poly-pyrrole implanted with AuNPs against SARS-CoV-2 Nucleocapsid (N) protein. The detection limits for the globular and nanotubular formulations were 7.442 and 0.4 ng/mL, respectively. As such, when adopting a nano-scale form, the nanotubular morphology showcased 8-fold higher sensitivity than its globular counterpart [97]. Furthermore, the nanotubular biosensor was tested against serum from positive and negative COVID-19 patients, where it successfully distinguished between all tested samples within 1 h. Given the small volume of serum needed to obtain a result, finger-prick blood tests for COVID-19 diagnosis and immunosurveillance could be a promising translation of this technology in a clinical setting [97].

With the rising rates of SARS-CoV-2 mutants, nanoparticle-based methods may provide novel detection methods or improve upon current methods to maintain high sensitivity and selectivity rates. Through an immunosensor utilizing MNPs conjugated to a mixture of antibodies, Durmus at al. were able to obtain higher detection rates of SARS-CoV-2 variants when compared to commercially available anti-SARS-CoV-2 S1 (anti-S1) and anti-S2 monoclonal antibodies [98]. The device’s LOD ranged from 0.53–0.75 ng/mL as opposed to the anti-S1 LOD of 0.93 ng/mL and the anti-S2 LOD of 0.99 ng/mL. The performance of the three immunosensor platforms were tested using 50 RT-PCR confirmed clinical samples (nasopharyngeal swabs; 40 positive and 10 negative). The positive results were further sub-classified according to the virus’s variant: original, alpha, beta and delta. The MNP immunosensor had a sensitivity and selectivity of 100% while the anti-S1 and anti-S2 platforms had sensitivity/selectivity values of 90%/100% and 87.5%/100%, respectively. To this end, the developed device showcased better ability to separate positive and negative samples with higher rates of variant detection. Nonetheless, it should be stated that the mixture of antibodies is derived from human samples, a method that is relatively unsustainable and has limit potential of mass production [98]. An alternative method of variant detection could be aptamer-based biosensing, an approach successfully demonstrated by Ellipilli et al., where DNA aptamer targeting a variant’s S protein is conjugated to AuNPs. [99] The aptamer-AuNP complex is then used as a detection probe, subsequently exhibiting a red color change when binding pre-anchored spike-antibodies on a nitrocellulose membrane. The aforementioned experiment served as a proof of concept, where the authors hope that such a method could be adopted to rapidly (<5 min) detect variant SARS-CoV-2 strains by cloning respective antibodies and customizing aptamers for mutated spike proteins [99]. Further, nanoparticles have been shown to markedly expediate the process of quantitative PCR, allowing for point-of-care application. Using gold nanorods, Blumenfeld et al. demonstrated plasmonic thermocycling through irradiation of the nanoparticles to facilitate rapid heating of a sample [100]. The incorporation of gold nanorods led to quenching of the overall fluorescence signal. However, the advantages gained are arguably worth the loss in fluorescence: the sample-to-result time was reported to be <25 min while still maintaining an LOD of 2.2–4.4 copies/μL and sensitivity/specificity values of 100%/100% from either saliva or nasal samples [100].

In addition to the detection of genetic material, antigens and antibodies against SARS-CoV-2, nanoparticles have also been applied to detect reactive oxygen species (ROS) and volatile organic compounds (VOCs) attributable to COVID-19. Using an electrochemical sensor decorated with Multi-Wall Carbon Nanotubes (MWCNTs) on its electrodes, Miripour et al. [101] were able to detect ROS in sputum samples. In its three-electrode conformation (working, reference and counter electrodes), changes in electrode potential were monitored using cyclic voltammetry. When validated with results from CT scans and RT-PCR, the device’s sensitivity/specificity results were 92%/94% and 97%/91% when compared to healthy individuals, respectively. Indeed, 97% of true positive patients were identified within 30 s. However, it needs to be noted that other respiratory conditions, such as asthma, acute pneumonia, etc., can also increase ROS [101]. Therefore, the device should not be used as a definitive diagnostic tool but rather as a rapid, assistive screening technique. In a paper revolving around a similar concept, Shan et al. [102] describe their multiplexed nanomaterial-based sensor array used to detect COVID-19 patients using their breath, where each array consists of 8 AuNPs linked to organic ligands. As a COVID-19 patient breathes into the device, the VOCs would react with the AuNPs, resulting in several detectable electrical signals. Through a case-control study design involving 49 confirmed COVID-19 patients, 58 healthy individuals and 33 non-COVID lung infection controls, the device exhibited an accuracy of 94% (training data set: 70% of the total sample) and 76% (test data set: 30% of the sample) in distinguishing COVID-19 patients from controls. Further, it had an accuracy of 90% (training data set) and 95% (test data set) in distinguishing between COVID-19 patients and other lung infection patients [102]. Presented in Table 1 is a summary of the main discussed nanoparticle modalities for the diagnosis of MERS-CoV, SARS-CoV and SARS-CoV-2.

## 6. Conclusions

The spread of COVID-19 has caused massive repercussions to the world’s healthcare system. Without any specific therapeutic procedures for COVID-19, proper knowledge of the virus’s structural and genomic components is required to develop alternative methods of combating the virus. One such method is using efficient diagnostic techniques as a preventative approach for the outbreak. RT-PCR and CT scan are used concomitantly as the standard procedure for diagnosing COVID-19 due to their accuracy and efficacy; however, several disadvantages have been noted for the aforementioned techniques, including false-negative results, high cost of operation and requirement of trained personnel. With the rise of SARS-CoV-2 variants and mutations that both worsen prognosis and complicate virus detection, challenges obstructing rapid, reliable and inexpensive COVID-19 diagnosis continue to emerge. As such, NPs have been identified in this review as possible candidates to cover the fallacies of RT-PCR and CT in the diagnosis of COVID-19.

This review has showcased the main studies revolving around the potential diagnostic utility of NPs in the COVID-19 pandemic. Considering the flexibility of NPs, they can be utilized either as standalone or supporting devices to current methods such as RT-PCR. Particularly, AuNP, AgNP, carbon nanotubes and graphene sheets have displayed promising capability to be used as a basis of a biosensor. AuNP has also portrayed great results when applied in lateral flow immunoassays. Moreover, superparamagnetic NPs can aid in RT-PCR by efficiently extracting RNA for the desired application. Given the ability of NPs to produce precise results within a relatively short period of time, NP-based diagnostic studies for SARS-CoV, MERS-CoV and SARS-CoV-2 can be utilized to better the available strategies currently used in the COVID-19 pandemic.

## Figures and Tables

**Table 1:** Highlights of nanoparticles/nanotechnologies-based diagnostic methods for coronaviruses.

Virus	Nanoparticle (NP) Type	Limit of Detection (LOD)	Mechanism of Detection	Qualities and Significance
MERS-CoV (79)	Gold NPs	1.0 pg/mL	Electromechanical immunosensor: Detection of voltametric response using an array of carbon electrodes modified with a gold NP coating.	Rapid; 20 min for results Lower LOD compared to ELISA (1 ng/mL)
MERS-CoV (80)	Gold NPs	1.0 pmol/µL	Colorimetric assay: in the absence of MERS-CoV targets, the gold NPs aggregate together and produce a color change	Rapid; 10 min for results Result is easy to interpret and can be seen with the naked eye Can detect virus with little-to-no amplification
MERS-CoV (82)	Silver NPs	1.53 nM	Colorimetric sensor: silver NPs used as colorimetric agent where color change is associated with the aggregation of NPs	High selectivity for desired oligonucleotides Can detect virus with little-to-no amplification Low-cost and disposable
SARS-CoV (85)	Gold NPs	1.0 pg/mL	Localized surface plasmon coupled fluorescence (LSPCF) fiber-optic biosensor: detection of fluorescence of a fluorophore-labeled viral target (N protein)	Quantitative: linear response seen between fluorescence signal and concentration of viral target (0.1 pg/mL to 1.0 ng/mL) LOD is 104-fold more sensitive than conventional ELISA Easy to use and disposable
SARS-CoV (86)	Gold NPs	2.5 pmol/L	Electrochemical genosensor: enzymatic amplification of hybridization signal	Rapid; 20 min for results
SARS-CoV (88)	Silica-coated superparamagnetic NPs	2.0 × 10^3^ copies	NPs used to capture cDNA targets for amplification. Amplified cDNA (via PCR) was separated from PCR products using NPs. Functionalized NPs were subsequently used as signaling probes and fluorescence was measured	Able to isolate desired cDNA targets from samples that contain both target cDNA and non-target cDNA Can be used as an adjuvant to PCR
SARS-CoV-2 (77)	Gold NPs	0.18 ng/µL	Colorimetric bioassay: in the presence of the N gene of SARS-CoV-2, the gold NP would aggregate, changing their surface plasmon resonance and precipitating	Rapid; 10 min for results Result can be seen with the naked eye Selective; no cross-reactivity with MERS-CoV Affordable and does not require sophisticated equipment
SARS-CoV-2 (90)	Gold NPs	2.29 fM for throat swab 3.99 fM for serum	Graphene field-effect transistor sensor: by targeting the RdRp genes, electrical changes on the sensor chip can be monitored	Near-perfect agreeability with RT-PCR (92%) Extremely rapid; <2 min for results
SARS-CoV-2 (94)	Gold NPs	-	Lateral flow immunoassay: able to detect both IgM and IgG antibodies against SARS-CoV-2. Result is portrayed via detection lines on the strip	Rapid; 15 min for results High selectivity and sensitivity; tested 525 clinical samples Easy to use and does not need additional equipment
SARS-CoV-2 (96)	Magnetic NPs (Iron NPs)	0.084 nM	Magnetic particle spectroscopy: binding of mimic SARS-CoV-2 and functionalized NPs lead to a change in magnetic response within an AC magnetic field	Rapid; <15 min for results Sensitive and selective Low cost and easy to handle
SARS-CoV-2 (101)	Multi-wall carbon nanotubes	-	Electrochemical sensor: detection of reactive oxygen species in sputum samples. Changes in electrode potential was measured using cyclin voltammetry	High selectivity and sensitivity Extremely rapid; 30 s till results Effective screening tool: cannot independently diagnose as reactive oxygen species can be alleviated by other respiratory conditions (e.g., asthma, acute bacterial pneumonia, etc.)

## Data Availability

Not applicable.
